# Monitoring changes in behaviour from multi-sensor systems

**DOI:** 10.1049/htl.2014.0089

**Published:** 2014-12-15

**Authors:** James D. Amor, Christopher J. James

**Affiliations:** Warwick Engineering in Biomedicine, School of Engineering, University of Warwick, Coventry CV4 7AL, UK

**Keywords:** behavioural sciences computing, patient monitoring, sensor fusion, medical signal processing, multisensor systems, behavioural patterns, health status, automated behaviour-monitoring systems, data analysis method

## Abstract

Behavioural patterns are important indicators of health status in a number of conditions and changes in behaviour can often indicate a change in health status. Currently, limited behaviour monitoring is carried out using paper-based assessment techniques. As technology becomes more prevalent and low-cost, there is an increasing movement towards automated behaviour-monitoring systems. These systems typically make use of a multi-sensor environment to gather data. Large data volumes are produced in this way, which poses a significant problem in terms of extracting useful indicators. Presented is a novel method for detecting behavioural patterns and calculating a metric for quantifying behavioural change in multi-sensor environments. The data analysis method is shown and an experimental validation of the method is presented which shows that it is possible to detect the difference between weekdays and weekend days. Two participants are analysed, with different sensor configurations and test environments and in both cases, the results show that the behavioural change metric for weekdays and weekend days is significantly different at 95% confidence level, using the methods presented.

## 1 Introduction

Behavioural change is an important factor in the maintenance of health and well-being in a number of conditions; a change in a person's behaviour can often be an indication of a change in their health state. Monitoring behaviour and detecting change as part of self-management can therefore be an effective tool for the maintenance of stable health in chronic diseases [1], including bipolar disorder [2] and chronic fatigue syndrome [3]. By monitoring behaviour, changes that correlate to prodromes or early warning signs can be identified and corrective or preventative action taken early. There are many paper-based solutions available for these conditions, but the prevalence of technology and potential cost savings are creating a driver towards more automated behaviour-monitoring systems.

Behaviour-monitoring systems, which are increasingly used in dementia care [4], typically take the form of distributed sensing environments that capture a large volume of information about the subject from a variety of sensors, such as in [5]. This produces a very large, multivariate dataset, often with significant elements of noise. The establishment of what constitutes a ‘normal’ pattern of behaviour and the detection of deviations from such a dataset pose significant challenges.

In this Letter, a novel approach to the detection of both normal behavioural patterns and deviations from these patterns is presented, which operates on a distributed sensing environment. The approach centres on the application of an existing algorithm, the continuous profile model (CPM), to an application domain it has not been previously applied to and the novel processing of the output of the CPM algorithm to detect behavioural patterns and changes in a multi-sensor system. This approach is termed multivariate behavioural modelling (MBM). An experimental validation of the MBM approach is presented to demonstrate that behavioural patterns and change can be detected by showing that the method is capable of determining the difference in behaviour between weekday and weekend days in two normal controls under different circumstances.

The method developed to detect change is described in Section 2, the experimental approach to validating the method is described in Section 3 and the results obtained are given in Section 4. Section 5 provides a discussion of the results and Section 6 presents some conclusions.

## 2 Method

The MBM method uses the CPM algorithm to detect the underlying behaviour from the data provided by the sensors in the system and to assess the similarity between new data and the established pattern for each sensor. The output from this step is then fused in an automatically weighted manner to detect the changes in behaviour.

The CPM has been chosen for this task as it is specifically designed to extract the underlying pattern from a cohort of input patterns and assumes that these patterns are all noisy and derived from some underlying pattern. This maps very closely to the problem addressed in this work of detecting an underlying behaviour pattern from several days of observation of free living behaviour.

The CPM is particularly suited to this task as it is cohort based, in that it works on the entire input set at once, and is capable of performing two key aspects of the analysis – identification of normal behavioural patterns and calculation of differences using the same model. Alternative methods exist that can handle one of these tasks, but not both. Dynamic time warping (DTW) [6], for example, is suited to analysing the difference between a known pattern and a new pattern, but does not provide a robust way to determine the underlying pattern. Statistical methods, such as a temporal average, could be used to perform this task but are less resistant to noise than the CPM. Other approaches such as identifying clusters of sensor firings as significant events and looking for the presence or absence of these [7] have been presented in the literature but do not offer the same granularity of output as the CPM.

This Section gives first an overview of the MBM method and then further details on the pattern detection and change detection algorithms.

### 2.1 Overview

The MBM method operates on a multivariate input set where several sensors exist that each produce one or more data streams. The MBM method proceeds in four steps:

(1) *Pre-processing*. The pre-processing step performs feature extraction on the raw data to compress the data stream into a set of characterising features. Pre-processing is not performed on data that are very simple, such as those from PIR sensors, which simply comprise the timestamps of sensor firings. Further details on potential pre-processing techniques can be found in [8]; however, a range of pre-processing options are possible dependent on the data being used.
(2) *Time-series generation*. A time-series generation step converts the pre-processed sensor output into a time series. At this point, the data stream has some level of basic meaning with respect to time and provides the lowest level of information in the system. An example of this is the processing of raw accelerometry data through to providing a measure of activity intensity within a five-minute epoch and generating a data stream consisting of activity scores for successive five-minute epochs. The pre-processing and time-series generation steps are bespoke for each data stream included in the MBM analysis. More details on suitable processing techniques can be found in [9].
(3) *Data fusion and pattern detection*. The data fusion and pattern detection step utilises the CPM algorithm to detect the normal pattern of activity from each time-series data stream and combines these to establish a normal pattern of behaviour for the user.
(4) *Change detection*. The change detection step uses the CPM algorithm to detect differences between the detected normal pattern and new data and fuses the detected difference scores across all data streams to detect deviations from the normal pattern of behaviour.

### 2.2 Pattern detection and change detection

The data fusion and pattern detection step is where the user's data are processed to extract their normal behaviour pattern. This pattern is used to detect changes in their behaviour over time. The core algorithms for both of these stages are in the CPM [10], which is a model for the simultaneous alignment, and estimation of the underlying pattern, of a set of time-warped input time series. Briefly, the CPM takes a set of repeated observations of a phenomenon, aligns them in time and generates a model for the underlying phenomenon that is capable of generating the input set. A full explanation of the CPM can be found in [10] but the principal points are repeated here as the change detection step makes use of a number of outputs that the CPM provides.

(1) *CPM background*. The primary assumption of the CPM is that there is an input set comprising many uniformly sampled time series that are all time-shifted and locally time-warped observations of an underlying process, termed the latent trace. This is analogous to a set of data recorded from a person repeating the same patterns of behaviour over many days; the underlying behaviour is the same, but individual parts may be shifted in time or take longer or shorter times to complete. The CPM provides a way to reverse engineer the input set to model the latent trace and align the input set in time.

Given a model for a particular input set, the CPM is capable of producing a log-likelihood of a new input being generated from the model in addition to the total log-likelihood of producing all the time series in the input data set from the model. These two measures, as well as the latent trace and time-aligned input set are the outputs of the CPM that are used by the MBM method.

(2) *MBM method*. The CPM is used in the MBM system to model the behaviour patterns for each data stream given a set of training data from that data stream. Assuming *N* data streams, the processing for each data stream is as follows. Over a training period of *T* days, the CPM model is trained and the total log-likelihood of producing the input set is calculated as *L_s_*, where *s* denotes the data stream. The average log-likelihood of generating any one of the input time series is thus calculated as
  $$L\alpha _s = \displaystyle{{L_s } \over T}$$

The latent trace obtained as a result of the training process corresponds to the detected normal pattern of behaviour observed for that data stream. During training a further measure, *ρ_s_*, is calculated as the correlation of the input set after alignment with the CPM. This is a measure of the consistency of the input from the data stream and is used in subsequent processing.

The process of pattern detection from training data is repeated for all the data streams in the system. In this way, a collection of known behavioural patterns is built up and collectively termed an activity signature. The activity signature defines the normal patterns of behaviour for the user as captured by the data streams used to monitor that user.

Once an activity signature is defined for a user, it is possible to calculate a metric that identifies how different a given day is to the activity signature. This calculation is performed in three steps. First, for each data stream, a likelihood measure for generating the new data from the optimised model is obtained using the CPM and denoted *Lβ_s_*. The weighted behavioural difference (WBD) between the new data and the established pattern can be calculated by comparing the difference between the two log-likelihoods as

$$W_s = \rho _s \lpar L\beta _s - L\alpha _s \rpar \; $$

where the multiplication by the consistency factor, *ρ_s_*, emphasises the differences in data streams that produce highly consistent data since a change in an otherwise consistent data source is deemed to be more significant than a change in an inconsistent data source. Furthermore, if any *W_s_* is positive it is set to 0, which has the effect of ignoring it in subsequent processing.

Given difference scores across all the data streams, an averaged WBD (AWBD) score for a single day can be calculated as

$$W_A = \displaystyle{1 \over N}\mathop \sum \limits_{s = 1}^N W_s $$

The averaging over *N* allows the calculation of *W_A_* to take account of a varying number of data streams and for a sensor to drop out without adversely distorting *W_A_*. The reasoning for ignoring positive *W_s_* values also becomes apparent in this equation. A positive *W_s_* indicates that the new data is highly typical of the training data; that it is not different. Since the objective is to identify differences, and since high positive scores could overwhelm smaller negative scores (which indicate difference), positive *W_s_* values are ignored.

By following the processing outlined above, a number of different data streams are used to calculate a single metric that quantifies how similar the behaviour of a new day is to the patterns that have been detected in the training data.

## 3 Technical trial

To develop and test the MBM method, data from a technical trial of a self-management system for bipolar disorder [8] was used. The trial was run at two sites using multi-sensor systems to monitor the behaviour of two participants, both health controls. Participant #1's trial lasted for 12 weeks and participant #2's trial lasted for 5 weeks. A slightly different sensor set was used in each trial and these are shown in Tables 1 and 2. Data obtained from the trials were used to develop the processing architecture described above.

**Table 1:** Sensor locations for participant #1

Sensor name	Location
camera	kitchen
PIR 1	bedroom
PIR 2	lounge
door switch 1	kitchen
door switch 2	kitchen
IR sensor	lounge
pressure mat	bedroom
environmental node	lounge

**Table 2:** Sensor locations for participant #2

Sensor name	Location
camera	kitchen
PIR 1	hallway
PIR 2	hallway
pressure mat	bedroom
environmental node	lounge

To test the MBM method, a hypothesis was made that the weekday behavioural patterns are different from weekend behavioural patterns and the system was used to detect this difference. For each trial participant, an activity signature was obtained based on their weekday behavioural data over a training period. Following the training period, the activity signature was used to assess the similarity of each new day. This was performed on both weekdays and weekends and the AWBD scores were used to identify weekend days from the data.

During the training period, only good days of data were used. A ‘good’ day of data is defined as a day with enough data that the processing algorithms run correctly. In practical terms, this means that when a sensor drops out during the training period, the training period for the data streams associated with that sensor are extended. This results in some of the data streams not being used at the start of the testing phase because they are still being trained. This is not a problem as the way the AWBD is calculated accounts for differing number of data streams.

## 4 Results

The results of the technical trial for participants #1 and #2 are presented in this Section. To take account of the difference in days of the two datasets, each set was analysed using a different number of days for training; participant #1's data was analysed with a 30-day training period and participant #2's data with a 7-day training period. A summary of the results is shown in Table 3.

**Table 3:** Summary of technical trial results

Participant	Trial length, weeks	Training length, days	*P*-value
#1	12	30	0.0182
#2	5	7	0.0106

### 4.1 Participant #1

**Figure 1:**
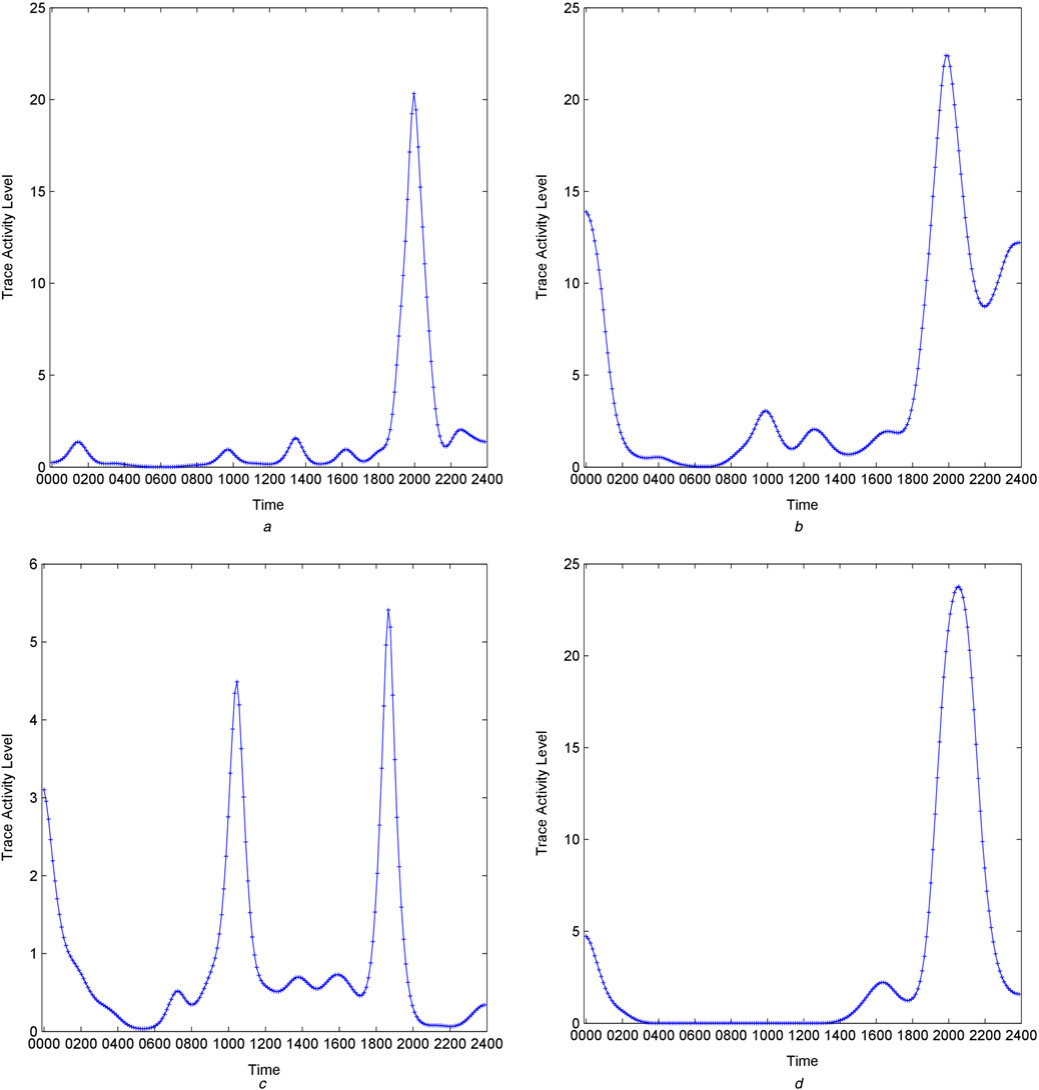
Behavioural traces from four data streams for participant #1 showing camera cooker AOI, PIR sitting room, pressure mat and environmental artifical light *a* Camera cooker AOI *b* PIR sitting room *c* Pressure mat *d* Environmental artificial light

Fig. 1 shows behavioural patterns obtained from four of the data streams from participant #1. The Camera Cooker AOI, Fig. 1*a*, shows a definite peak at around 2000 with smaller peaks during the day. The PIR sitting room pattern, Fig. 1*b*, and environmental artificial light pattern, Fig. 1*d*, also show activity peaks around this time and the PIR pattern continues to identify activity later into the evening. The pressure mat pattern, Fig. 1*c*, identifies three peaks, around 1000, 1900 and 0000.

**Figure 2:**
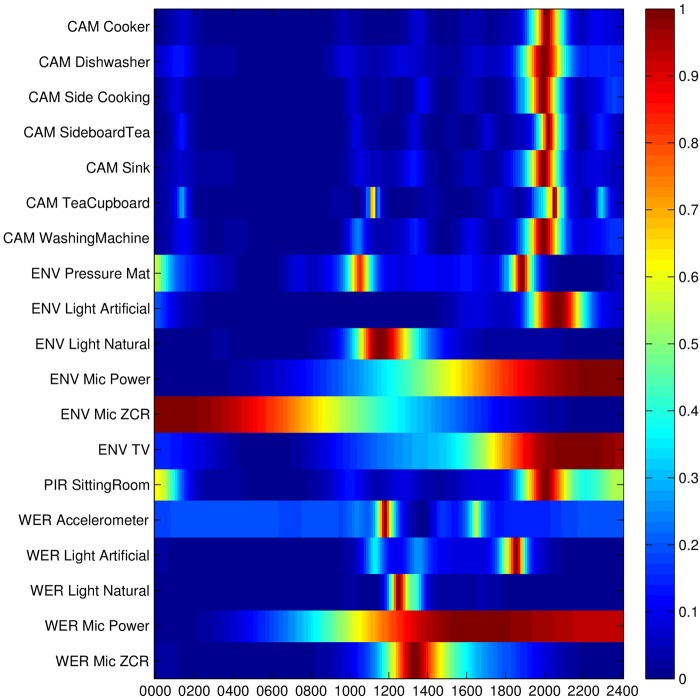
Activity signature for participant #1 showing how behavioural patterns from each data stream align in time

The overall activity signature for participant #1 is shown in Fig. 2, in which amplitudes of the behavioural patterns have been normalised for display purposes. The behavioural patterns from Fig. 1 can be seen from this figure along with behavioural patterns from the remaining sensors. The alignment between the behavioural patterns can be clearly seen from this figure, in particular the activity in the evening, around 2000, and during the middle of the day, around 1300.

The activity peaks identified in Figs. 1 and 2 are consistent with the general patterns known for participant #1, particularly leaving the office for lunch and preparing food in the evening. This is particularly evident in the data streams ‘WER Accelerometer’ and ‘CAM Cooker’.

**Figure 3:**
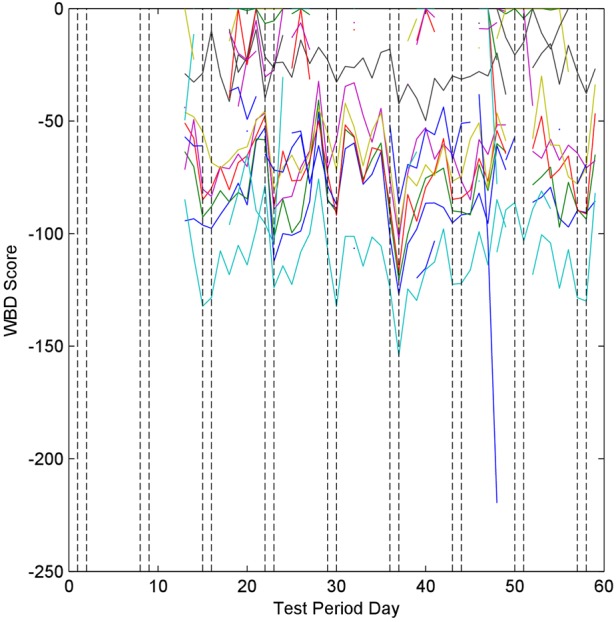
WBD scores for all data streams for participant #1

Fig. 3 shows all of the WBD scores from the test data. Gaps in the data are because of sensor problems and missing data. In general, it is difficult to identify any trends from this graph; however, there are several days that show a drop across several sensors, notably days 23, 30 and 37.

**Figure 4:**
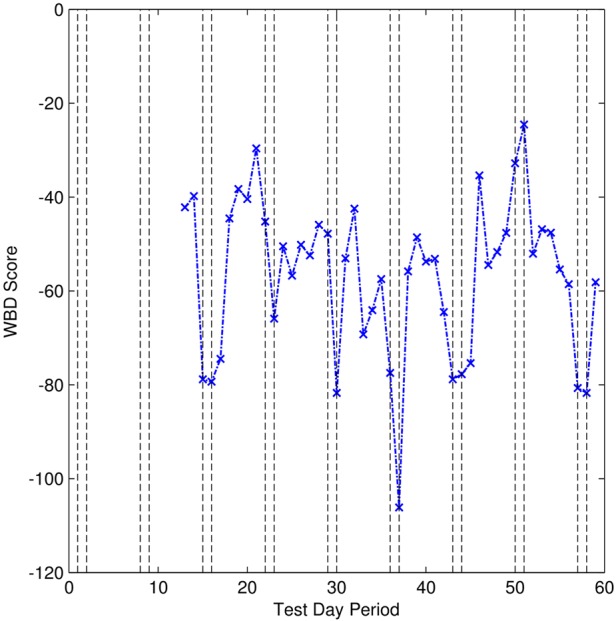
AWBD scores obtained from participant #1

Fig. 4 shows the AWBD score over the testing period for participant #1. From this figure, the difference between weekdays and weekends is much clearer; weekends have a significantly lower AWBD score than weekdays. This observation is verified with a Student's *t*-test on the data with a null hypothesis that there is no statistical difference between the scores obtained on weekdays and weekends. A *P*-value of 0.0182 was obtained, which rejects the null hypothesis at the 5% confidence interval and indicates that there is a significant difference between weekday and weekend AWBD scores for participant #1.

### 4.2 Participant #2

**Figure 5:**
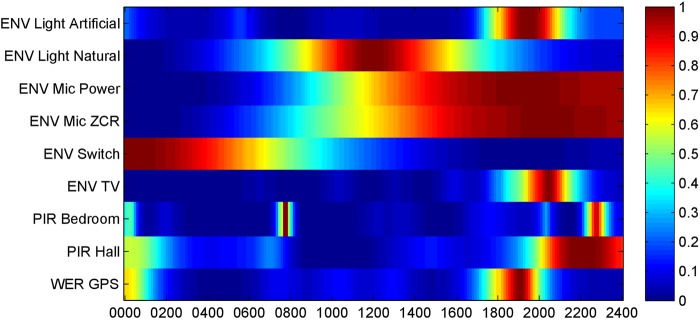
Activity signature for participant #2 showing how behavioural patterns from each data stream align in time

Fig. 5 shows the activity signature for participant #2. There is no particular correlation in the data streams, other than a trend for activity in the evenings. Only one sensor, the PIR, is of particular note as it displays two distinct activity peaks, one in the morning and one in the evening. These correspond to the times that participant #2 got up and went to bed.

**Figure 6:**
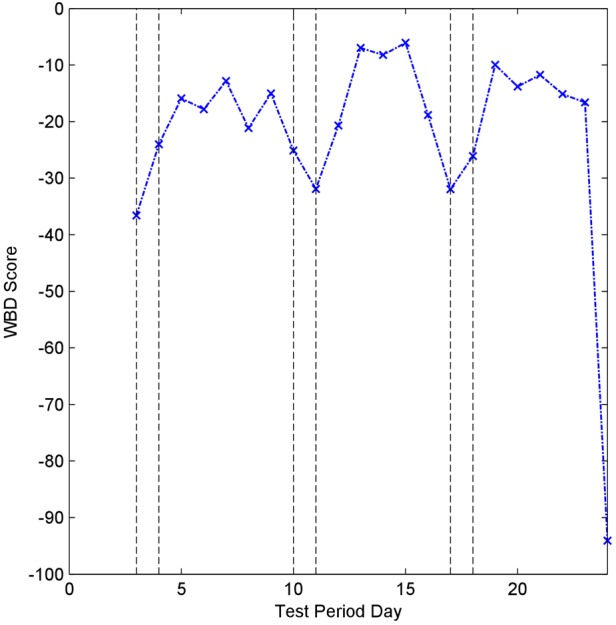
AWBD scores obtained from participant #2

Fig. 6 shows the AWBD score for the test period from participant #2. As with participant #1, the weekends can be clearly seen as different. This is verified with a Student's *t*-test *P*-value of 0.0106 which rejects the null hypothesis at the 5% significance level and indicates that there is a significant difference between the weekday and weekend AWBD scores.

Table 3 shows the *t*-test results for both participants. Both tests reject the null hypothesis (that there is no statistical difference between the scores obtained on weekdays and weekends) at the 5% significance level.

## 5 Discussion

The results of the technical trial show that behavioural patterns can be extracted and that the difference between weekdays and weekend days can be identified; the AWBD scores were shown to be significantly different at the 95% confidence level for both participants. The AWBD metric allows easy identification of difference and allows an inspection of the individual WBD scores to identify those that caused a difference to be flagged, providing additional insight into the user's behaviour. Furthermore, the results show that the calculation of the AWBD metric is able to run successfully with poor quality data that include significant and inconsistent gaps across different data streams.

The data processing does not identify every weekend as different, but it would not be expected to. It is entirely possible that the behavioural pattern for a weekend day might be similar enough to a weekday for it to be given the same score. A record of what each participant did on each day of the trial was not maintained, which prevented a complete verification that the change detection algorithm was working correctly for each day. However, a general record of activity for each participant was maintained and this is sufficient to know that, in general, they behaved differently at the weekend and the Student's t-tests confirm that the AWBD scores in the weekend and weekday groups are statistically different.

It is, however, acknowledged that there are some misclassifications present in the data. A principal cause of some of these in the testing period is sensor drop-out. If the sensors that are important in distinguishing between weekdays and weekends drop-out then the system will not be able to distinguish between the two. Days 50 and 51 from participant #1 display this type of misclassification and can be seen in Fig. 3. Many of the sensors dropped out over those 2 days, causing a reduction in the number of data streams available for classification. Of the data streams remaining, two were light sensors, which report the same pattern each day. Thus, the WBD scores for the light sensor data streams are very high, resulting in a very high AWBD score for those days.

The robustness of the algorithm to this form of missing data, and to noisy data comes from two factors. Noisy data are accounted for through the use of the probabilistic approaches in the CPM and through the consistency factor, which deliberately reduces the influence of noisy data. Robustness to missing data is a result of the modular treatment of the difference scores from each data stream. By treating each data stream independently and averaging scores as the final step, missing days of data do not adversely affect the AWBD unless those sensors are key to determining the behavioural difference, as can be seen for days 50–51 for participant #1.

## 6 Conclusions

The work presented in this Letter demonstrates an approach to monitoring behavioural change from multi-sensor systems and shows that macro-level changes in behaviour can be identified using the MBM method. The use of a cohort-based pattern detection method (CPM) in this application to generate activity signatures, and the use of automatically weighted behavior difference scores in the data fusion step are the principal novel contributions of this work. In particular, the way in which the data are analysed provide for a method that is particularly robust to noise and data drop-out and that can adjust automatically to the addition and removal of sensor components.

The results, presented in summary form in Table 3, show that the AWBD scores for weekdays and weekend days are significantly different at the 95% confidence level for two different test scenarios, providing *P*-values of 0.0182 and 0.0106, respectively, which strongly reject the null hypothesis that there is no difference between weekday and weekend data. Behavioural differences are shown in both participant's data and this indicates that the MBM method is capable of operation on differing datasets with differing underlying behavioural characteristic expressed by the two trial participants.

The above results show that the MBM method provides a solid foundation for detecting behavioural change and a single overarching indicator metric that can be drilled down into to provide additional insight. This approach could be used as a data analysis technique in self-management systems for chronic health as a means of detecting changing behaviour patterns and further work with this approach will focus on this area.

In such a system, the input sensor devices could be tailored to be condition specific and a decision support system (DSS) used to provide insight to the user. The DSS would be able to take action when behaviour change was detected, such as providing alerts or suggestions to the user. Since the data processing can be examined to identify specific changes, these, coupled with the use of specific input sensors, could be used to make inferences about the user's health state, predicting affective change, for example, in bipolar disorder.

The development of such a system has the potential to provide a significant improvement in long-term health for people with chronic conditions.

Additionally, the work in this Letter could be extended to support the detection of different patterns for different days, such as weekend or weekday patterns and an automation of this process could be investigated with a cluster-based approach on the activity signatures. There would also be utility in developing a mechanism to identify the specific aspects of a day that were different from the normal, both in terms of which sensors are different, but more importantly, to localise those differences in time. These improvements would allow for greater flexibility in behaviour detection and provide a more targeted and specific indicator of change.

## References

[1] Thomas Bodenheimer M.D. Kate Lorig R.N. Halsted Holman M.D. Kevin Grumbach M.D.: ‘Patient self-management of chronic disease in primary care’, J. Am. Med. Associat., 2002, 288, (19), pp. 2469–2475 (doi: ) 10.1001/jama.288.19.2469 12435261

[2] Basco M.R. Rush A.J.: ‘Cognitive-behavioral therapy for bipolar disorder’ (Guilford Press, 1996)

[3] Nijs J. Paul L. Wallman K.: ‘Chronic fatigue syndrome: an approach combining self-management with graded exercise to avoid exacerbations’, J. Rehabilitation Med., 2008, 40, (4), pp. 241–247(7) (doi: ) 10.2340/16501977-0185 18382818

[4] Lotfi A. Langensiepen C. Mahmoud S.M. Akhlaghinia M.J.: ‘Smart homes for the elderly dementia sufferers: identification and prediction of abnormal behavior’, J. Ambient Intell. Humanized Comput., 2012, 3, (3), pp. 205–218 (doi: )

[5] James C.J. Crowe J. Magill E.: ‘Personalised ambient monitoring (PAM) of the mentally ill’. In Fourth European Conf. of the Int. Federation for Medical and Biological Engineering, Vol. 22, 2009

[6] Berndt D.J. James C.: ‘Using dynamic time warping to find patterns in time series’. KDD workshop, 1994, Vol. 10, No. 16, pp. 359–370

[7] Barger T.S. Brown D.E. Alwan M.: ‘Health-status monitoring through analysis of behavioral patterns’, IEEE Trans. Syst. Man Cybern. A, Syst. Humans, 2005, 35, (1), pp. 22–27 (doi: )

[8] Amor J.D. James C.J.: ‘Behavioral pattern detection from personalized ambient monitoring’. Proc. of the 32nd Annual Int. Conf. of the IEEE EMBS, Buenos Aires, Argentina, September 2010 10.1109/IEMBS.2010.5626102 21095823

[9] Amor J.D.: ‘Detecting and monitoring behavioural change through personalised ambient monitoring’ (University of Southampton, 2011)

[10] Listgarten J. Neal R.M. Roweis S.T. Emili A.: ‘Multiple alignment of continuous time series’. Advances in Neural Information Processing Systems 17, Cambridge, MA, USA, 2005, pp. 817–824

